# PTPRM methylation induced by FN1 promotes the development of glioblastoma by activating STAT3 signalling

**DOI:** 10.1080/13880209.2021.1944220

**Published:** 2021-07-05

**Authors:** Jian Song, Di Zhao, Guozhu Sun, Jiankai Yang, Zhongqiang Lv, Baohua Jiao

**Affiliations:** a Department of Neurosurgery, The Second Affiliated Hospital, Hebei Medical University, Shijiazhuang, China; b Department of Neurosurgery, The First Affiliated Hospital, Hebei Medical University, Shijiazhuang, China

**Keywords:** GBM cells, proliferation, p-STAT3, DNA methylation

## Abstract

**Context:**

The phosphorylation of signal transducer and activator of transcription protein 3 (STAT3) is up-regulated in glioblastoma (GBM) cells and is regulated by protein tyrosine phosphatase receptor type M (PTPRM). Fibronectin-1 (FN1) is also reported to be up-regulated in GBM.

**Objective:**

We explored the role of FN1-induced PTPRM methylation in GBM.

**Materials and methods:**

The lentivirus particles of oe-PTPRM, sh-PTPRM, oe-FN1, sh-FN1, or their negative controls (NSCs) were transfected into GBM cells with or without stattic (0.5 μM, 24 h) or 5-aza (1 μM, 0, 2, 4 h) treatments. Methylation-specific PCR was performed to detect PTPRM methylation levels.

**Results:**

PTPRM was down-regulated (0.373 ± 0.124- and 0.455 ± 0.109-fold), FN1 and p-STAT3 were up-regulated (*p* < 0.001) in A172 and U87 MG cells as compared to NSCs. Overexpressing PTPRM inhibited STAT3 phosphorylation. Interfering with PTPRM increased colony numbers in A172 and U-87 MG cells (2.253 ± 0.111- and 2.043 ± 0.19-fold), and stattic reduced them. Cell viability was reduced after treatment with 5-aza in A172 and U-87 MG cells (*p* < 0.05). P-STAT3 was down-regulated after 5-aza treatment. Overexpressing FN1 decreased PTPRM levels (*p* < 0.001), knockdown of FN1 decreased PTPRM methylation and inhibited STAT3 phosphorylation. Overexpressing FN1 increased cell viability (1.497 ± 0.114- and 1.460 ± 0.151-fold), and stattic or 5-aza reversed such effects (*p* < 0.05).

**Discussion and conclusions:**

The up-regulation of FN1 reduced PTPRM by increasing its methylation, resulting in an increase of STAT3 phosphorylation and promoting GBM cell proliferation. Interfering with FN1 may be a potential therapeutic target for GBM.

## Introduction

Glioblastoma, also known as glioblastoma multiforme (GBM), is the most aggressive primary brain neoplasm and accounts for 15% of all brain tumours (Bleeker et al. 2012; Ostrom et al. 2014). Most patients survive less than a year after diagnosis, and despite maximum treatment, cancer usually recurs (Bleeker et al. 2012). The prognosis for GBM patients is dismal, and the median survival time is only 15 months (Marenco-Hillembrand et al. 2020; Yang et al. 2020). The aetiology of most GBM cases remains unknown, and there are no effective preventive measures. Furthermore, most current clinical treatments cannot completely eradicate all tumour cells (Gallego 2015). Therefore, it is necessary to further study the molecular mechanism of the occurrence and development of GBM to help in its diagnosis and treatment.

Signal transducer and activator of transcription protein 3 (STAT3) is a transcription factor associated with the occurrence, invasion, and metastasis of tumours (Germain and Frank 2007). It is known that the phosphorylation of STAT3 is up-regulated in GBM cells and affects further proliferation (Newman et al. 2017). Protein tyrosine phosphatase receptor type M (PTPRM) belongs to the protein tyrosine phosphatase (PTP) family, members of which have been reported to have a potential tumour inhibition effect (Tonks 2006). Meanwhile, it has been reported that the overexpression of the PTP family of molecules (DUSP26 or PTPRT) in E98 GBM cells decreases the incidence of tumours (Bourgonje et al. 2016). PTPRM is a member of the PTP family that targets the STAT3 pathway, and its overexpression has been shown to directly reduce the phosphorylation of STAT3 Y705 (Im et al. 2020). PTPRM has been reported as a mutated tumour-associated factor in many cancers. However, whether PTPRM can affect the occurrence and development of GBM by regulating STAT3 phosphorylation remains to be seen.

Epigenetic changes are very important in the development of GBM (Kozono et al. 2015). Among the different epigenetic modifications, DNA methylation remains the most intensively studied. It is known that O^6^-methylguanine-DNA methyltransferase (MGMT) promoter methylation has prognostic and predictive value for GBM patients (Hegi et al. 2004, 2005). In addition, DNA methylation has been reported to regulate GBM cell proliferation by silencing anti-fibrosis genes (Kirstein et al. 2020). These findings have therapeutic implications because epigenetic changes are reversible. Stevenson et al. (2014) indicated that protein tyrosine phosphatase receptor type K methylation in acute lymphoblastic leukaemia is significantly associated with STAT3 phosphorylation. However, whether PTPRM methylation is associated with STAT3 phosphorylation in GBM remains unknown.

Fibronectin is a kind of glycoprotein in the extracellular matrix that binds to other extracellular matrix proteins to regulate a variety of cellular activities (Mao and Schwarzbauer 2005). Fibronectin plays an important role in cell adhesion, growth, migration, and differentiation and is essential for wound healing and embryonic development. The up-regulation of fibronectin expression is related to primary malignancies (Vasaturo et al. 2005). In addition, fibronectin has been reported to be increased in many tumours, such as in mammary tumorigenesis and lung cancer (Han et al. 2006; Williams et al. 2008). Previous studies have found that fibronectin-1 (FN1) is up-regulated in GBM cells and may be an important key gene (Long et al. 2017). Therefore, this paper intends to explore whether FN1 regulates STAT3 phosphorylation through PTPRM methylation and further influences the proliferation of glioma cells.

## Materials and methods

### Cell culture and transfection

Human GBM cell lines A172 and U-87 MG and neural stem cells (NSCs) obtained from ATCC (Manassas, VA, USA) were cultured in DMEM (Thermo Fisher, Waltham, MA, USA) containing 10% foetal bovine serum (Thermo Fisher) and 1% penicillin/streptomycin (Thermo Fisher, USA). The cells were placed at 37 °C in a humid incubator with 5% CO_2_ (Xu et al. 2018).

For cell transfection, overexpressing PTPRM (oe-PTPRM), short hairpin RNA against PTPRM (sh-PTPRM), overexpressing FN1 (oe-FN1), short hairpin RNA against FN1 (sh-FN1), sh-FN1 + sh-PTPRM, or their negative controls (NCs) (GeneChem Co. Ltd, Shanghai, China) were constructed into lentiviral vectors and transfected into 293 T cells for 72 h to establish lentivirus particles. Then the lentivirus particles were transfected into the A172 and U-87 MG cells. The cells were then cultured in an incubator at 37 °C with 5% CO_2_ for 48 h.

### Quantitative real-time PCR (qRT-PCR)

Total RNA was extracted from the A172, U-87 MG, and NSCs using TRIzol reagent (Invitrogen, Shanghai, China) and reverse transcribed into single-stranded cDNA (PrimeScript^™^ reagent Kit, TaKaRa, Japan). The obtained cDNA was then mixed with SYBR Green PCR Master Mix (TaKaRa, Japan) and amplified on an ABI 7900 fast thermocycler (Applied Biosystems, Thermo Fisher, USA). The primers were synthesized by Sangon Biotech (Shanghai, China). Relative expression was calculated using the 2^-ΔΔCt^ method and GAPDH served as an internal control. All experiments were carried out in triplicate (Liu et al. 2020; Wang et al. 2020; Zhang et al. 2020).

The primer sequences were as follows:

FN1: (F: 5′-GAGAATAAGCTGTACCATCGCAA-3′

R: 5′-CGACCACATAGGAAGTCCCAG-3′);

PTPRM: (F: 5′-AAGAGACCATGAGCAGCACC-3′

R: 5′-ATCACCATCTTCCAGGAGCGA-3′);

GAPDH: (F: 5′-CCTTCTCCATGGTGGTGAAGAC-3′

R: 5′-AATGAGAAAGCCTCGTCGCA-3′)

### Western blot

Proteins from the A172, U-87 MG, and NSCs were extracted with a RIPA lysis buffer (Beyotime, Shanghai, China). The lysates were then centrifuged at 12,000 rpm for 20 min at 4 °C. The concentration of the proteins was examined via a BCA protein assay kit (Thermo Fisher, USA). The proteins were then isolated using SDS-PAGE and transferred to a polyvinylidene difluoride membrane (Merck Millipore, Germany). Afterwards, the membrane was blocked with 5% skimmed milk for 1 h at room temperature, followed by incubation with primary antibodies against FN1 (ab2413, 1:1000), PTPRM (CST, 1:1000), STAT3 (ab68153, 1:1000), p-STAT3 (ab76315, 1:2000), and β-actin (ab8227, 1:1000) at 4 °C overnight. The membrane was washed with tris-buffered saline Tween-20 and then incubated with HRP-conjugated antibody (CST, 1:1000) for 2 h at room temperature and visualized with ECL Plus Western Blotting Substrate (Thermo Fisher).

### Colony formation assay

The A172 and U-87 MG cells treated with sh-PTPRM, sh-NC, or stattic (STAT3 inhibitor) were cultivated in 6-well culture plates for 2 weeks. The colonies were fixed with 4% paraformaldehyde for 15 min and stained with 0.5% crystal violet (Sigma-Aldrich, Germany) for 15 min.

### 5-Aza-2′-deoxycytidine (5-aza) treatment

The A172, U-87 MG, and NSCs were cultured with 5-aza (1 μM, Sigma-Aldrich) for 0, 2, and 4 h. The RNA and proteins were extracted for the following detection.

### Methylation-specific PCR (MSP)

Genomic DNA was extracted from the A172, U-87 MG, and NSCs using the QIAGEN DNeasy Extraction System (Qiagen, Hilden, Germany). The quantity of DNA was measured by NanoDrop 2000 (Thermo Scientific). Sodium sulphite was then added to the DNA samples. After purifying through the reaction column, the acquired DNA was used for the subsequent PCR. The primers were designed by Sangon Biotech (Shanghai, China). The acquired PCR products were electrophoresed with agarose gel and visualized under UV light.

### 3-(4,5-Dimethylthiazol-2-yl)-2,5-diphenyltetrazolium bromide (MTT) assay

Cell proliferation was detected by MTT assay. The A172 and U87 MG cells were treated with 5-aza (1 μM) for 4 h, and the 5-aza was then washed, and the samples were cultured in a new medium for 48 h. The survival rate was then measured using an MTT reagent (M5655, Sigma-Aldrich). After 48 h incubation, 10 μL of MTT solution (5 mg/mL) was added to the cells, and the samples were incubated for a further 3 h at 37 °C. The supernatant was removed and dimethylsulphoxide (Sigma-Aldrich) was added to dissolve the formazan crystals. Absorbance was determined at the 450 nm wavelength.

### Statistical analysis

All experiments were repeated in at least triplicate. Data were presented as means ± standard deviations, and statistical analyses were conducted using SPSS 21.0 software (SPSS Inc., USA). Differences between the two groups were determined using a Student’s *t*-test, while a one-way ANOVA was used for more than two groups. *p*-Values <0.05 were regarded as statistically significant (Roman et al. 2020).

## Results

### PTPRM was down-regulated in GBM cells

**Figure 1.**
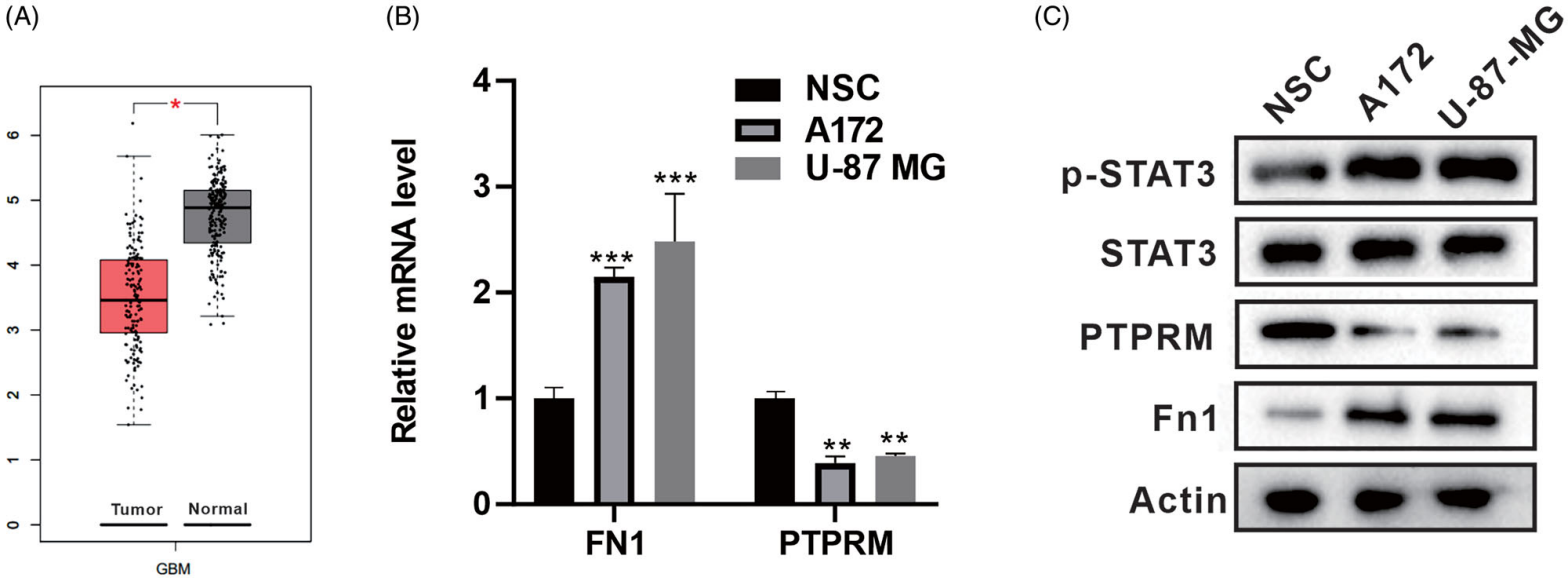
PTPRM was down-regulated in GBM cells. (A) Bioinformatics analysis with GEPIA was performed to detect PTPRM expression in GBM. (B) The mRNA levels of FN1 and PTPRM were quantified by qRT-PCR. (C) The protein levels of FN1, PTPRM, and p-STAT3 were detected by western blot. *p < 0.05 vs. Normal. **p < 0.01 vs. NSC. ***p < 0.001 vs. NSC.

First, we conducted a bioinformatics analysis with GEPIA (http://gepia2.cancer-pku.cn) (Tang et al. 2017) and found that PTPRM expression was down-regulated in GBM cells (Figure 1(A)). The mRNA levels of FN1 and PTPRM in the A172, U-87 MG, and NSCs were then determined, and the results showed that FN1 was up-regulated in the A172 (2.148 ± 0.086 vs. 1 ± 0.06, *p* < 0.001) and U87 MG (2.582 ± 0.138 vs. 1 ± 0.06, *p* < 0.001) cells as compared to the NSCs, and PTPRM was down-regulated in A172 (0.373 ± 0.124 vs. 1 ± 0.036, *p* < 0.01) and U87 MG (0.455 ± 0.109 vs. 1 ± 0.036, *p* < 0.01) cells as compared to the NSCs (Figure 1(B)). Figure 1(C) showed that the expression of FN1 and p-STAT3 in the A172 and U87 MG cells was increased and PTPRM was decreased as compared to the NSCs.

### PTPRM and phosphorylation of STAT3 levels were associated with the proliferation of GBM cells

**Figure 2.**
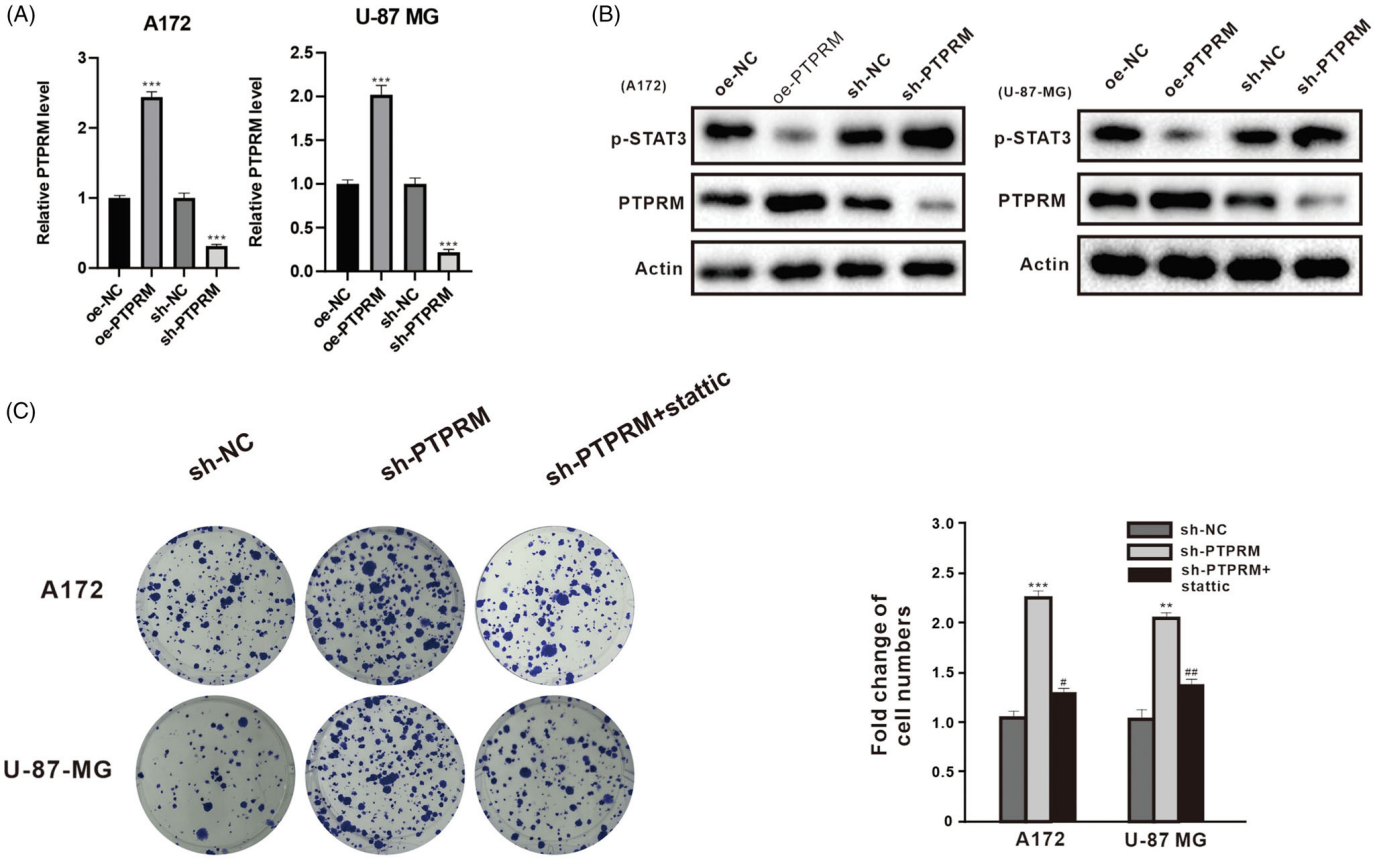
PTPRM and phosphorylation of STAT3 levels were associated with the proliferation of GBM cells. A172 and U-87 MG cells were transfected with oe-PTPRM or sh-PTPRM and their negative controls (NC). (A) The mRNA level of PTPRM was quantified by qRT-PCR. (B) The protein levels of PTPRM and p-STAT3 were detected by western blot. (C) A172, U-87 MG cells were treated with sh-PTPRM or sh-NC or stattic (STAT3 inhibitor). The colony formation assay was used to detect and compare the cell cloning formation. **p < 0.01 vs. sh-NC. ***p < 0.001 vs. sh-NC or oe-NC. ^#^*p* < 0.05 vs. sh-PTPRM. ^##^*p* < 0.01 vs. sh-PTPRM.

To determine the impact of PTPRM on the proliferation of GBM cells, the lentivirus particles of oe-PTPRM, sh-PTPRM, or their NCs were transfected into the A172 and U-87 MG cells. The qRT-PCR results indicated that PTPRM was up-regulated in the oe-PTPRM group in the A172 (2.44 ± 0.075 vs. 1 ± 0.06, *p* < 0.001) and U-87 MG (2.02 ± 0.105 vs. 1 ± 0.08, *p* < 0.001) cells and down-regulated in the sh-PTPRM group in the A172 (0.313 ± 0.025 vs. 1 ± 0.08, *p* < 0.001) and U-87 MG (0.22 ± 0.03 vs. 1 ± 0.06, *p* < 0.001) cells (Figure 2(A)). Western blot also obtained a similar result, and the overexpression of PTPRM significantly inhibited STAT3 phosphorylation (Figure 2(B)). The A172 and U-87 MG cells were then treated with sh-PTPRM, sh-NC, or stattic (STAT3 inhibitor). Colony formation assay showed that the number of cells in the sh-PTPRM group increased in the A172 (2.253 ± 0.111 vs. 1 ± 0.062, *p* < 0.001) and U-87 MG (2.043 ± 0.19 vs. 1 ± 0.151, *p* < 0.01) cells, and then decreased after being treated with stattic in the A172 (1.370 ± 0.335 vs. 2.253 ± 0.111, *p* < 0.05) and U-87 MG (1.327 ± 0.146 vs. 2.043 ± 0.19, *p* < 0.01) cells (Figure 2(C)). The results indicated that PTPRM and the phosphorylation of STAT3 levels might be associated with the proliferation of GBM cells.

### DNA methylation affected PTPRM expression, the phosphorylation level of STAT3, and the proliferation of GBM cells

**Figure 3.**
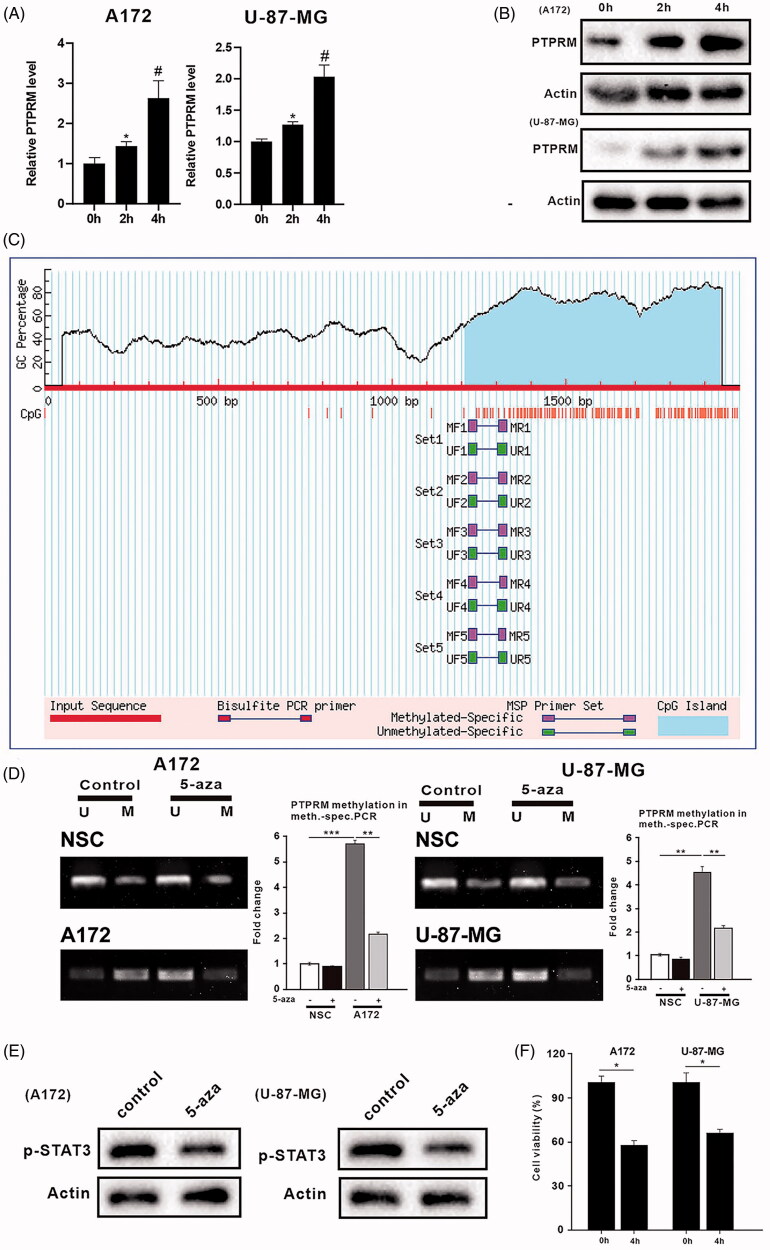
DNA methylation affected PTPRM expression, the phosphorylation level of STAT3, and proliferation of GBM. A172, U-87 MG, and NSCs were treated with 5-aza (1uM). (A) The mRNA level of PTPRM was quantified by qRT-PCR. (B) The protein level of PTPRM was examined by western blot. (C) The online analysis software (http://www.urogene.org/cgi-bin/methprimer/methprimer.cgi) was used to analyze the PTPRM promoter region. (D) MSP was performed to detect the PTPRM methylation levels. (E) STAT3 phosphorylation level was evaluated using western blot. (F) MTT assay was used to detect cell proliferation. *p < 0.05 vs. 0 h. **p < 0.01 vs. 5-aza (−). ***p < 0.001 vs. 5-aza (−). ^#^*p* < 0.05 vs. 2 h.

To explore the regulatory effect of DNA methylation on PTPRM, the phosphorylation level of STAT3, and the proliferation of GBM cells, A172, U-87 MG, and NSCs were treated with 5-aza (1 μM). The qRT-PCR and western blot results showed that PTPRM was continuously elevated in the A172 (2.631 ± 0.633 vs. 1 ± 0.05, *p* < 0.05) and U-87 MG (2.033 ± 0.387 vs. 1 ± 0.05, *p* < 0.05) cells, while PTPRM levels did not change significantly after being treated with 5-aza for 4 h in the NSCs (Figure 3(A,B), Supplementary Figure 1(B)). We then analyzed the promoter region of PTPRM and found that PTPRM had methylation sites (Figure 3(C)). Next, MSP was performed to detect the DNA methylation of PTPRM further. The results showed that the DNA methylation level of the PTPRM gene promoter was higher in the A172 (5.697 ± 0.25 vs. 1 ± 0.221, *p* < 0.001) and U-87 MG (4.42 ± 0.685 vs. 1 ± 0.062, *p* < 0.01) cells than in the NSCs. PTPRM methylation levels were down-regulated in the A172 cells (2.203 ± 0.662 vs. 5.697 ± 0.25, *p* < 0.01) and U-87 MG (2.173 ± 0.187 vs. 4.42 ± 0.685, *p* < 0.01) cells after treatment with 5-aza (Figure 3(D)). Figure 3(E) showed that STAT3 phosphorylation was inhibited after treatment with 5-aza (1 μM). To investigate the effects of 5-aza on cell proliferation after a methylation intervention, 5-aza was added to the A172 and U-87 MG cells for 4 h. The MTT assay results showed that cell survival was significantly reduced after the 5-aza treatment in the A172 (0.587 ± 0.135 vs. 1 ± 0.08, *p* < 0.05) and U-87 MG (0.673 ± 0.078 vs. 1 ± 0.095, *p* < 0.05) cells (Figure 3(F)). These findings illustrated that the expression of PTPRM, phosphorylation level of STAT3, and proliferation of GBM cells were all related to DNA methylation.

### FN1 regulated PTPRM expression through DNA methylation

**Figure 4.**
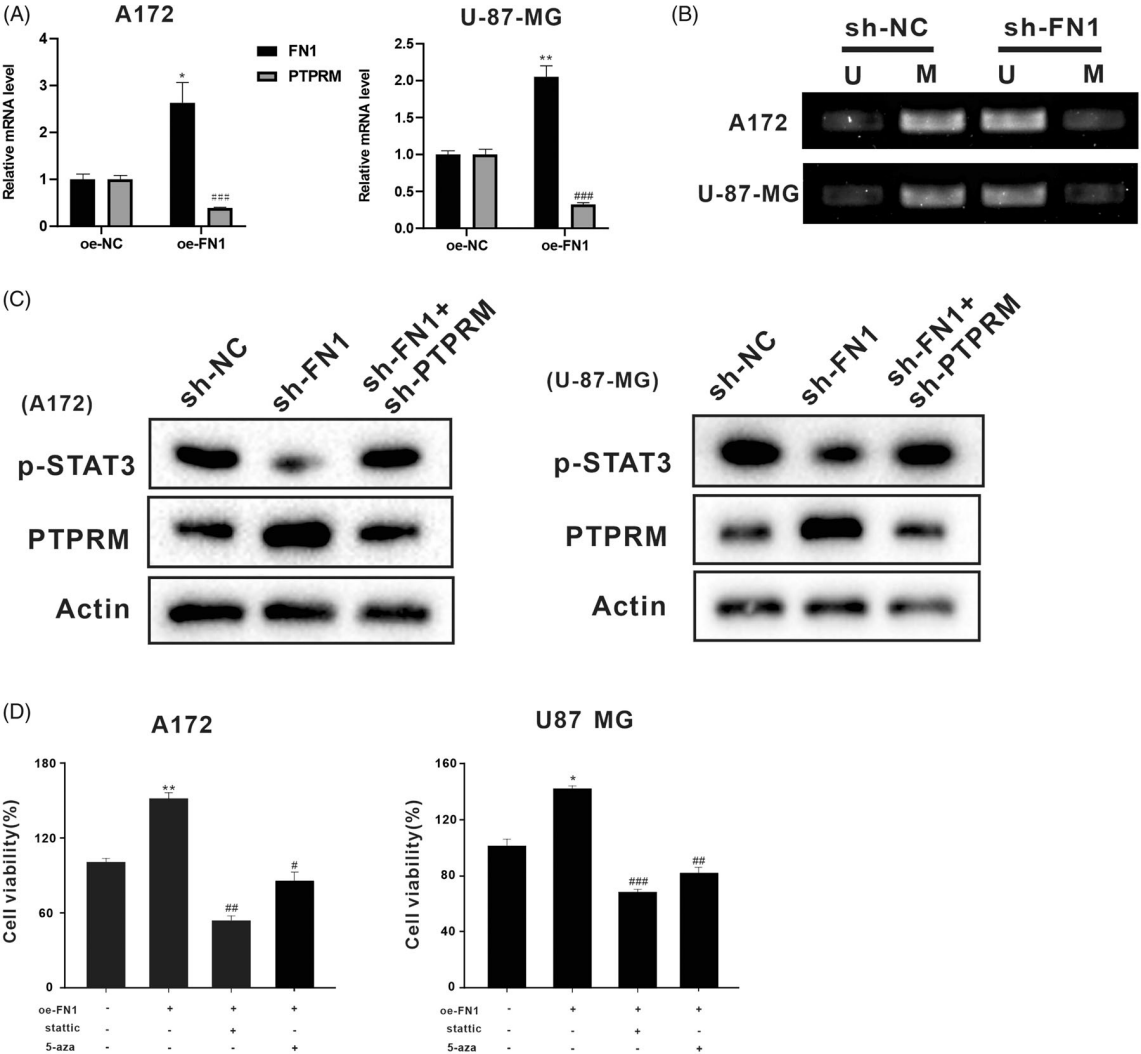
FN1 regulated PTPRM expression through DNA methylation. The oe-FN1, sh-FN1, and sh-FN1 + sh-PTPRM particles were transfected into A172 and U-87 MG cells. (A) The mRNA levels of PTPRM were detected by qRT-PCR. (B) MSP was performed to detect the PTPRM methylation levels after the knockdown of FN1. (C) Protein levels of PTPRM and STAT3 phosphorylation were determined by western blot. (D) Cell viability was detected by MTT assay. *p < 0.05 vs. Normal or oe-NC. **p < 0.01 vs. Normal or oe-NC. ^#^*p* < 0.05 vs. oe-FN1. ^##^*p* < 0.01 vs. oe-FN1. ^###^*p* < 0.001 vs. oe-FN1 or oe-NC.

In the following work, we investigated whether FN1 regulated PTPRM expression through DNA methylation. We inducted oe-FN1, sh-FN1, and sh-FN1 + sh-PTPRM in the A172 and U-87 MG cells. Figure 4(A) displayed the mRNA levels of PTPRM and FN1. The results showed that FN1 was increased in the A172 (2.631 ± 0.633 vs. 1 ± 0.07, *p* < 0.05) and U-87 MG (2.087 ± 0.204 vs. 1 ± 0.079, *p* < 0.01) cells after being transfected with oe-FN1. PTPRM was decreased in the A172 (0.392 ± 0.014 vs. 1 ± 0.092, *p* < 0.001) and U-87 MG (0.323 ± 0.024 vs. 1 ± 0.111, *p* < 0.001) cells after being transfected with oe-FN1. As compared to sh-NC, the methylation level of PTPRM was down-regulated after being transfected with sh-FN1 (Figure 4(B)). The cells were then divided into three groups: sh-NC, sh-FN1, and sh-FN1 + sh-PTPRM. The western blot results showed that PTPRM expression was increased and the phosphorylation level of STAT3 was decreased in the sh-FN1 group, while the effect was reversed after being co-transfected with sh-FN1 and sh-PTPRM (Figure 4(C)). The A172 and U-87 MG cells were then treated with stattic or 5-aza based on oe-FN1. Cell proliferation was increased when FN1 was overexpressed in the A172 (1.497 ± 0.114 vs. 1 ± 0.056, *p* < 0.01) and U-87 MG (1.460 ± 0.151 vs. 1 ± 0.09, *p* < 0.05) cells, while it was decreased after being treated with stattic (STAT3 inhibitor) in the A172 (0.567 ± 0.153 vs. 1.497 ± 0.114, *p* < 0.01) and U-87 MG (0.680 ± 0.036 vs. 1.460 ± 0.151, *p* < 0.001) cells or 5-aza in the A172 (0.80 ± 0.248 vs. 1.497 ± 0.114, *p* < 0.05) and U-87 MG (0.81 ± 0.08 vs. 1.460 ± 0.151, *p* < 0.01) cells (Figure 4(D)). These assays indicated that FN1 might regulate the expression of PTPRM through DNA methylation.

## Discussion

GBM is the most common tumour among central nervous system cancers and has a low survival rate (Jemal et al. 2011; Johnson and O’Neill 2012). However, few studies have been reported to explore the molecular mechanisms of GBM. In this study, we tested the hypothesis that the up-regulation of FN1 in GBM may reduce the expression level of PTPRM through DNA methylation, promoting STAT3 phosphorylation and the proliferation of GBM cells. The results showed that PTPRM was down-regulated in GBM cells, and the overexpression of FN1 increased PTPRM methylation, the phosphorylation of STAT3, and the proliferation of GBM cells.

STAT3 contains a conserved tyrosine residue at position 705 and can be activated by a variety of cytokines, growth factors, and other stimuli (Germain and Frank 2007). STAT3 has been found to be activated in many types of tumour cells, and STAT3 activation is associated with reduced tumour survival (Benekli et al. 2002; Alvarez et al. 2005). Furthermore, inhibitors of STAT3 activation may suppress the survival and the proliferation of tumour cells (Alas and Bonavida 2003). In addition, Birner et al. (2010) demonstrated that the activation of STAT3 by Y705 phosphorylation is related to clinically more aggressive GBM. It has also been reported that STAT3 phosphorylation is increased in GBM cells (Tonks 2006). Similarly, in our experiments, the phosphorylation level of STAT3 was also increased in A172 cells and U-87 MG cells (Figure 1(C)).

PTPRM is a member of the PTP family that targets the STAT3 pathway, and it is also a novel type of STAT3 PTP. A recent study demonstrated that the overexpression of PTPRM reduces STAT3 phosphorylation in lung cancer malignancies (Im et al. 2020). Through a bioinformatics analysis using GEPIA (http://gepia.cancer-pku.cn/index.html), PTPRM was found to be lowly expressed in GBM cells. The QRT-PCR and western blot analysis in cells from human GBM cell lines A172 and U-87 MG showed the same result. In addition, we constructed a lentivirus (OE - PTPRM/SH - PTPRM) that overexpressed and interfered with PTPRM to detect the effect of a PTPRM intervention on phosphorylation of STAT3 and the proliferation of GBM cells. The results showed that the overexpression of PTPRM decreased the phosphorylation of STAT3 and cell proliferation. In addition, STAT3 phosphorylation was increased in the sh-PTPRM group and the effect was reversed after interference with PTPRM and stattic treatment (Figures 2(B,C), Supplementary Figure 1(A)).

It is known that epigenetic changes such as DNA methylation will affect gene expression and cell function. MGMT methylation has been reported to be significantly associated with the prognosis of glioma patients treated with temozolomide (Bell et al. 2018). In our study, we analyzed the PTPRM promoter region through online analysis software (http://www.urogene.org/cgi-bin/methprimer/methprimer.cgi), and we were surprised to find that there were methylation sites for PTPRM. MSP assay indicated that PTPRM methylation was reduced and STAT3 phosphorylation was inhibited in A172 and U-87 MG cells after being treated with 5-aza. Meanwhile, cell survival was also significantly reduced after 5-aza treatment. The results indicated that PTPRM methylation may be a potential diagnostic and therapeutic biomarker for glioma patients.

Fibronectin 1 (FN1) is a type of adhesive glycoprotein that is highly expressed in many tumour cells (gastric, thyroid, etc.). Furthermore, a high abundance of FN1 has been detected in both MT330 and LN229 GBM cell lines (Yang et al. 2019). Studies have also shown that fibronectin can affect the expression of MMP2 through methylation (Pereira et al. 2014). Meanwhile, its extracellular matrix has also been reported to inhibit the expression of apoptotic regulatory factor Fas through methylation (Thaler et al. 2010). In this study, the results suggested that the PTPRM methylation level was decreased after interference with FN1, and STAT3 phosphorylation was inhibited at the same time. The overexpression of FN1 decreased the expression of PTPRM and increased cell proliferation. The data also indicated that FN1 might reduce the expression of PTPRM through DNA methylation and further promote STAT3 phosphorylation and cell proliferation.

## Conclusions

The results of the present study have first demonstrated that a low expression of PTPRM promotes cell proliferation by increasing STAT3 phosphorylation in GBM. In addition, we have also demonstrated for the first time that FN1 can induce the methylation of PTPRM.

## Supplementary Material

Supplemental Material
