# Supplementary figures and images for: PTPRM methylation induced by FN1 promotes the development of glioblastoma by activating STAT3 signalling
Source: Pharm Biol. 2021 Jul 5;59(1):902–9. doi: 10.1080/13880209.2021.1944220 (PMC8259858; doi:10.1080/13880209.2021.1944220)

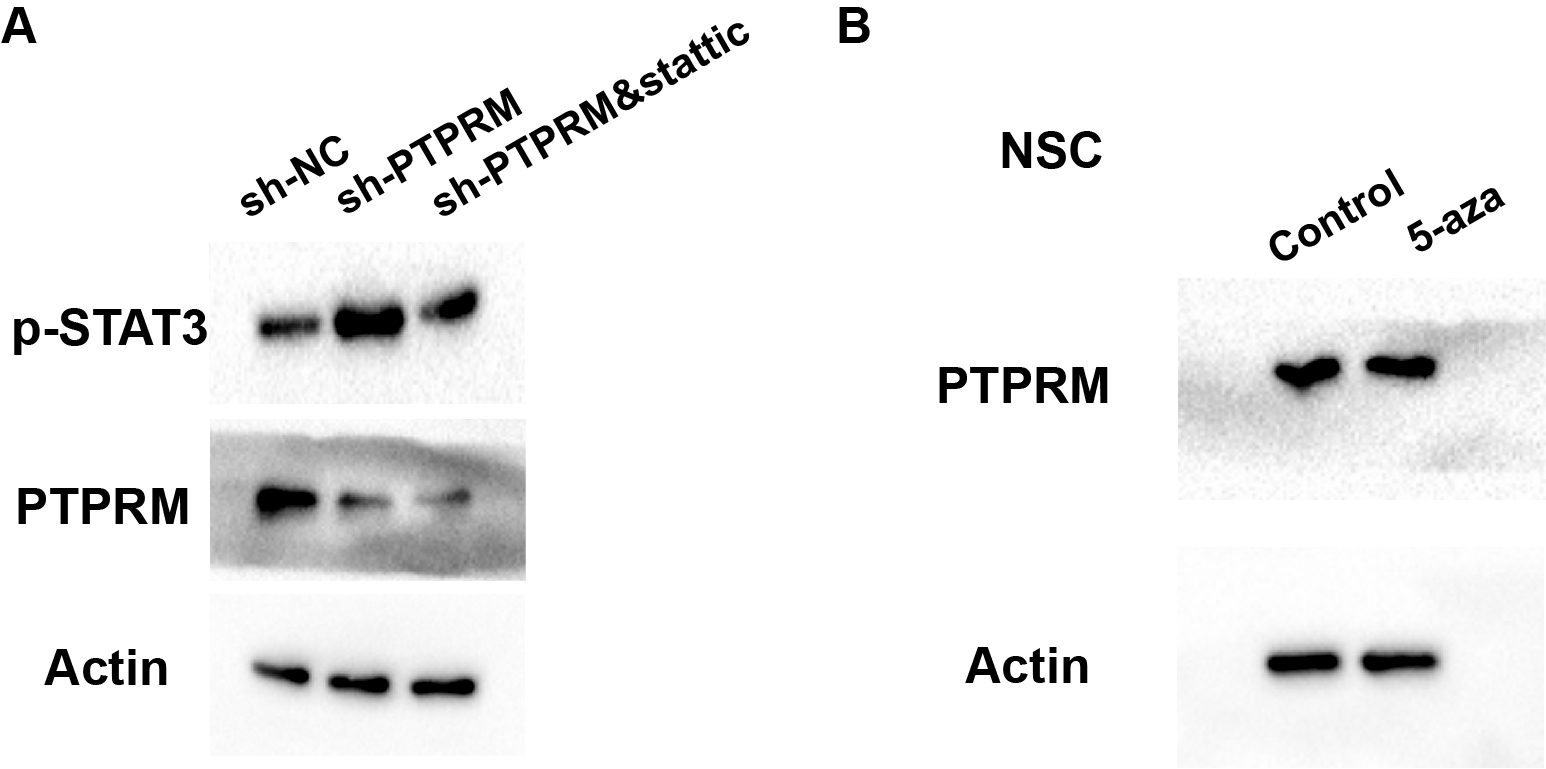

Supplement: Supplemental Material [file IPHB_A_1944220_SM9456.jpg]
